# Hyperphosphorylation of Tau Due to the Interference of Protein Phosphatase Methylesterase-1 Overexpression by MiR-125b-5p in Melatonin Receptor Knockout Mice

**DOI:** 10.3390/ijms222111850

**Published:** 2021-10-31

**Authors:** Han Zhao, Lingyan Feng, Wei Zhong, Hongyan Zhen, Qingjia Chi, Xiang Wang

**Affiliations:** 1 Department of Histology and Embryology, Medical College, Jianghan University, Wuhan 430030, China; zhaohan@mail.ustc.edu.cn (H.Z.); zhongwei@jhun.edu.cn (W.Z.); 2 Department of Immunology, Medical College, Jianghan University, Wuhan 430030, China; Fengly@jhun.edu.cn; 3 Department of Pathology and Pathophysiology, Medical College, Jianghan University, Wuhan 430030, China; zhenhy100@jhun.edu.cn; 4 Department of Mechanics and Engineering Structure, Wuhan University of Technology, Wuhan 430070, China; qingjia@whut.edu.cn

**Keywords:** melatonin, PME-1, mmu-miR-125b-5p, neurofibrillary tangles, protein phosphatase 2A, tau

## Abstract

Melatonin has been indicated to ameliorate tau hyperphosphorylation in the pathogenesis of tau diseases, but the role of melatonin-receptor signal transduction has not been clearly discovered. In this study, we found intensive tau hyperphosphorylation in melatonin receptor knockout mice. Bielschowsky silver staining showed ghostlike neurofibrillary tangles in melatonin receptor-2 knockout (MT2KO) as well as melatonin receptors-1 and -2 knockout (DKO) mice, and an argyrophilic substance was deposited in melatonin receptor-1 knockout (MT1KO) mice. Furthermore, we found significantly decreased activity of protein phosphatase 2A (PP2A) by Western blot and enzyme-linked immunosorbent assay (ELISA), which was partly due to the overexpression of protein phosphatase methylesterase-1 (PME-1), but not glycogen synthase kinase-3β (GSK-3β), cyclin-dependent kinase 5 (CDK5) or protein kinase B (Akt). Finally, we observed a significant increase in cyclic adenosine monophosphate (cAMP) and a decrease in miR-125b-5p levels in MT1KO, MT2KO and DKO mice. Using a luciferase reporter assay, we discovered that miR-125b-5p largely decreased the expression of firefly luciferase by interfering with the 3′UTR of PME-1. Furthermore, miR-125b-5p mimics significantly decreased the expression of PME-1, while miR-125b-5p inhibitor induced tau hyperphosphorylation. These results show that melatonin-receptor signal transduction plays an important role in tau hyperphosphorylation and tangle formation.

## 1. Introduction

Melatonin (N-acetyl-5-methoxytryptamine, MT) is widely found in almost all living organisms [1]. In vertebrates, and especially in mammals, melatonin is centrally synthetized by the pineal gland to work as a hormone, and its level is controlled by circadian rhythms [2]. Melatonin plays its role not only directly in non-receptor-mediated antioxidant activity [3,4,5], but also in receptor-mediated signal transduction pathways [6,7,8]. There are two types of melatonin receptors: melatonin receptor 1 (MT1) and melatonin receptor 2 (MT2) [9]. Both receptors are G-protein-coupled receptors that decrease cAMP, but there are some differences in their signal transduction [10]. The MT1 receptor transduces several cellular responses through both pertussis toxin-sensitive and toxin-insensitive pathways. Activation of the MT1 receptor through the Gi protein inhibits forskolin-stimulated cAMP formation, protein kinase A (PKA) activity, and phosphorylation of the cAMP-responsive element binding protein (CREB), and its activation through the Gq protein increases intracellular calcium [11]. Meanwhile, MT1-receptor activation stimulates c-Jun N-terminal kinase activity via both pertussis toxin-sensitive (Gi) and insensitive (Gs, Gz and G16) proteins [12]. The activation of recombinant MT2 receptors expressed in mammalian cells inhibits forskolin-stimulated cAMP formation and cyclic guanosine monophosphate (cGMP) accumulation and increases phosphoinositide hydrolysis. In COS-7 cells expressing the human MT2 receptor, melatonin induces c-Jun N-terminal kinase via pertussis toxin-sensitive (Gi) and toxin-insensitive (G16) proteins [13]. The activation of MT2 receptors inhibits GABA_a_ receptor-mediated function in the hippocampus and increases protein kinase C (PKC) activity in the rat suprachiasmatic nucleus (SCN) [14].

It is known that melatonin plays a major role in the human body. Studies have found that melatonin has several functions including anti-inflammatory [15,16,17], antioxidant [18,19], circadian rhythm and sleep [20,21], anticancer [22,23,24,25], aging [26,27], etc. Furthermore, melatonin can be used in several neurological diseases [28,29], including Alzheimer’s disease (AD) [30,31], Parkinson’s disease [32], multiple sclerosis [33,34], Huntington’s disease [35], epilepsy [36], depression [37], schizophrenia [38], and so on. From which, AD is an age-dependent neurodegenerative disease. Several studies have shown that melatonin levels are largely decreased, and circadian rhythms are disrupted in the early stage of Alzheimer’s disease [39]. Furthermore, expression of melatonin receptor 2 is obviously decreased, but expression of melatonin receptor 1 is increased in AD, indicating that melatonin signal transduction may be involved in AD pathology [40]. Interestingly, melatonin has been reported to mitigate neuronal tau hyperphosphorylation and cognitive deficit, but its mechanism is still unclear [41].

We used melatonin receptor knockout mice to explore melatonin-receptor function. Significantly phosphorylated tau was observed in three transgenic (MT1KO, MT2KO and DKO) mice, and even ghostlike neurofibrillary tangles formed in MT2KO and DKO mice. To further examine the mechanism for this, we detected the activity of a tau phosphorylation-related kinase. We found significantly decreased activity of PP2A due to the overexpression of PME-1. To explore dysfunction in melatonin signal transduction, we examined its downstream targets and found an increase in cAMP and a decrease in miR-125b-5p levels. Using a luciferase reporter assay, we confirmed that miR-125b-5p decreased PME-1 expression by interfering with its translation via a dramatic increase in the level of PME-1 mRNA. Finally, we also transfected the N2a cell lines with miR-125b-5p inhibitor and HEK293 cell lines with a miR-125b-5p mimic, and found the direct regulation of PME-1 by miR-125b-5p. Together, our results support the significant role of melatonin signal transduction on tau phosphorylation and tauopathy.

## 2. Results

### 2.1. Melatonin Receptor Knockout Mice Exhibit a Strong Signal for Hypophosphorylated Tau

**Figure 1:**
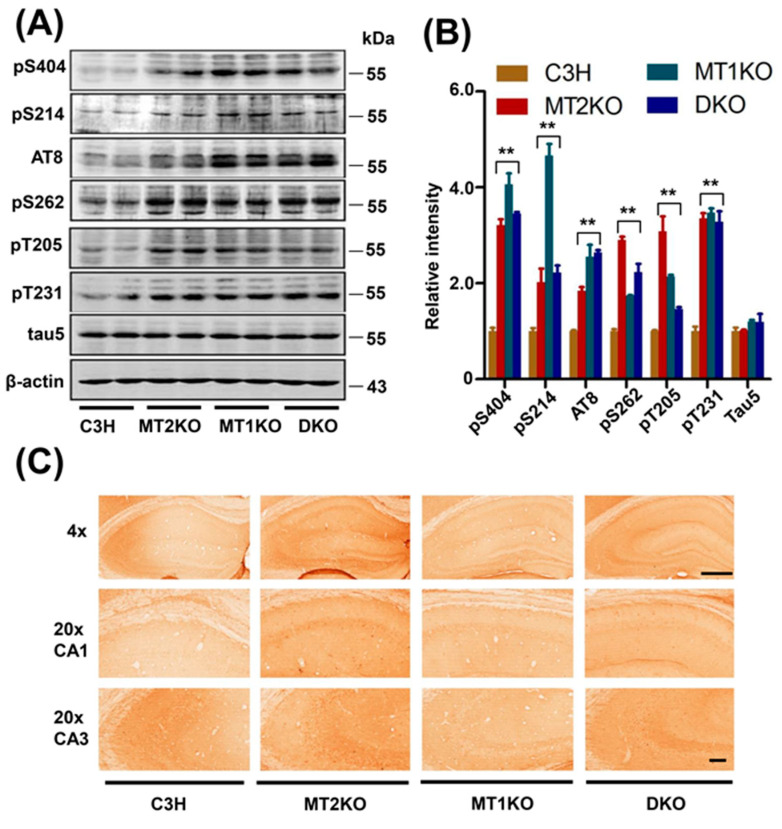
Melatonin receptor knockout mice exhibit tau hyperphosphorylation. (**A**) The Western blotting of pS404 (phosphorylated tau at Ser404), pS214 (phosphorylated tau at Ser214), AT8 (human PHF-tau), pS262 (phosphorylated tau at Ser262), pT231 (phosphorylated tau at Thr231), pT205 (phosphorylated tau at Thr205), tau 5 (total tau). (**B**) Relative quantification of pS404 (phosphorylated tau at Ser404), pS214 (phosphorylated tau at Ser214), AT8, pS262, pT231, pT205, tau5. (**C**) Immunohistochemistry for the tau phosphorylation at the site of serine 262. Bar = 100 μm. ** *p* < 0.01, compared with C3H. Data are presented as means ± SD.

**Figure 2:**
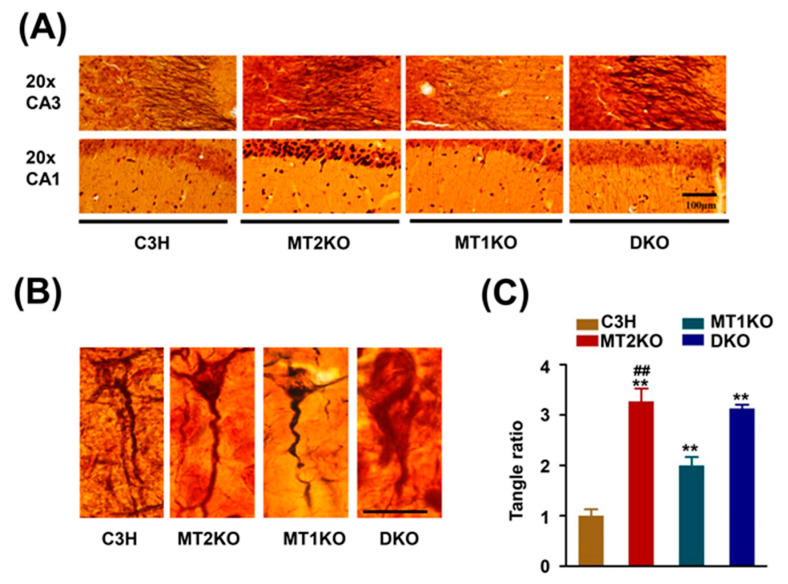
Bielschowsky silver staining. (**A**) Bielschowsky silver staining in hippocampus. (**B**) Bielschowsky silver staining for ghostlike neurofibrillary tangles in three transgenic mice. (**C**) Relative number for the total typical neuron in the four groups. Bar = 100 μm. ** *p* < 0.01, compared with C3H. ## *p* < 0.01, compared with MT1KO mice. Data are presented as means ± SD.

C3H, MT1KO, MT2KO and DKO hippocampus samples were incubated with the pS404, pS214, AT8, pS262, pT231, pT205, and Tau 5 antibodies to detect tau phosphorylation. As shown in Figure 1A,B, there was no significant difference in the total protein level but obvious phosphorylation at the pS404, pS214, AT8, pS262, pT231, and pT205 sites in three transgenic mice groups. Hyperphosphorylated pS262 was also found by immunohistochemistry (Figure 1C). Because hyperphosphorylated tau forms a β-folded complex and can be stained by Bielschowsky silver staining, we used this method to observe the phosphorylated tau distribution in the intracellular and extracellular space. Obvious changes in the distribution of folded proteins were uncovered by silver staining of the cortex and hippocampus (Figure 2A). C3H pyramidal cells exhibited a clean signal in a distinct outline. However, in the MT2KO mice, we observed ghostlike tangled aggregates at the axon hillock, and apical dendrites were curved. In the DKO mice, the apical dendrites were degenerated, and large neurofibrillary tangles were observed. In the MT1KO mice, the apical dendrites were also curved, and there was a small amount of black staining (Figure 2B). Finally, we randomly selected twenty sliver-stained slices and counted the cells and ghostlike tangles. As shown in Figure 2C, there were significantly increased ghostlike tangles and curved apical dendrites in slices from the MT2KO and DKO mice.

### 2.2. Tau Hyperphosphorylation Is Caused by the Decreased Activity of PP2A

**Figure 3:**
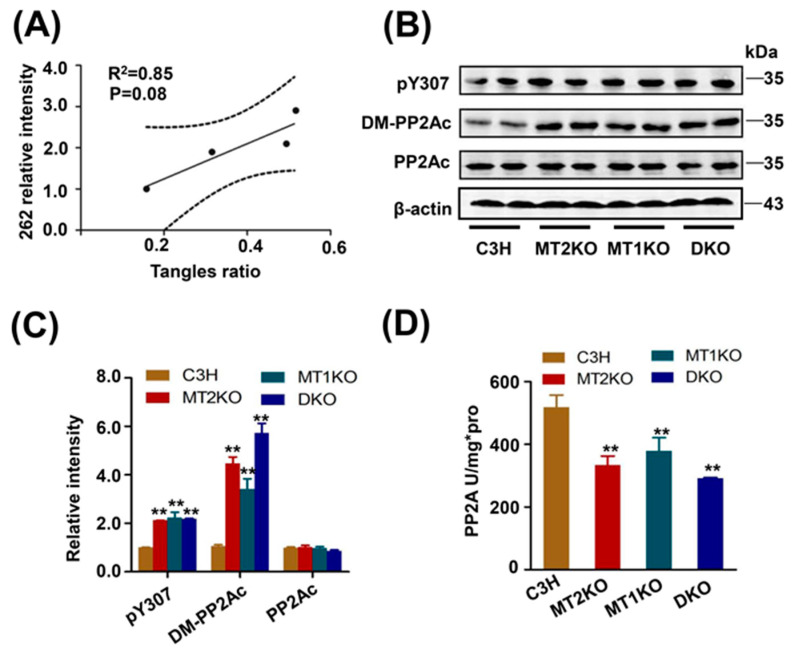
Decreased activity of PP2A in the three transgenic mice. (**A**) Linear regression conducted between pS262 and tangle ratio. (**B**) Western blotting of pY307-PP2Ac, DM-PP2Ac and PP2Ac. (**C**) Relative quantity of pY307-PP2Ac, DM-PP2Ac and PP2Ac. (**D**) Detecting the activity of PP2A by ELISA. ** *p* < 0.01, compared with C3H. Data are presented as means ± SD. PP2A: protein phosphatase 2A. DM-PP2Ac: demethylation of PP2Ac.

In order to confirm the correlation between tangles and tau phosphorylation, we analyzed the intensity of tau phosphorylation at several sites and tangle ratios by linear regression. The pS262 was strongly associated with tangles formation by linear regression (R^2^ = 0.85, *p* = 0.08) (Figure 3A), but not pS404 (R^2^ = 0.40, *p* = 0.37), pT231 (R^2^ = 0.57, *p* = 0.25), AT8 (R^2^ = 0.30, *p* = 0.45), or pT205 (R^2^ = 0.56, *p* = 0.25) (Supplementary Figure S1A–D). PP2A is the main dephosphorylated kinase for tau protein, especially at the site of Ser262. From Western blot analysis, we detected the demethylated and phosphorylated catalytic subunit of PP2A (PP2Ac) (Figure 3B) and found that both of them were increased, especially demethylated PP2Ac, but there was no difference in the total quantity of PP2Ac (Figure 3C). To confirm the PP2A activity, we used ELISA and found an obvious decrease in its activity in MT2KO, MT1KO and DKO mice compared with that in C3H mice (Figure 3D). Several kinases are involved in tau hyperphosphorylation. Among various serine/threonine kinases, CDK5, GSK-3β, Akt, and ERK are known to regulate tau phosphorylation. Their activities were contrary to tau phosphorylation in MT2KO, MT1KO and DKO mice (Supplementary Figure S2A–F). These results suggested that the decreased activity of PP2A is the cause of tau hyperphosphorylation.

### 2.3. The Melatonin Receptor Regulates PP2A through PME-1

**Figure 4:**
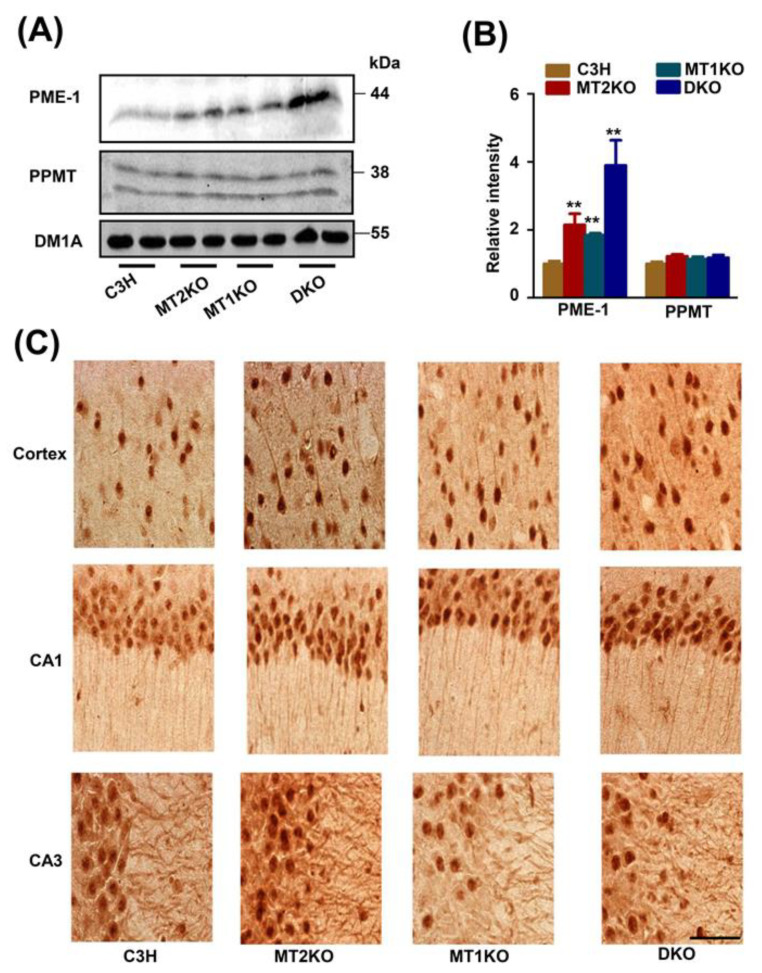
The expression of PME-1 in three transgenic mice. (**A**) Western blotting of PME-1 and PPMT. (**B**) Relative intensity of PME-1 and PPMT. (**C**) Immunohistochemistry for PME-1. Bar = 100 μm. ** *p* < 0.01, compared with C3H. Data are presented as means ± SD.

Because we detected high levels of the phosphorylated and demethylated PP2A in the three transgenic mice groups, we further analyzed protein phosphatase methylesterase (PME-1) and PP2A-methyltransferase (PPMT) levels, and found an increased quantity of PME-1 in three transgenic mice groups compared with their levels in the control mice (Figure 4A,B). Immunohistochemical experiments showed the dramatic deposition of PME-1 in both the nucleus and cytoplasm (Figure 4C).

### 2.4. cAMP Promotes the Transcription of PME-1 by Decreasing the Production of miR-125b-5p

**Figure 5:**
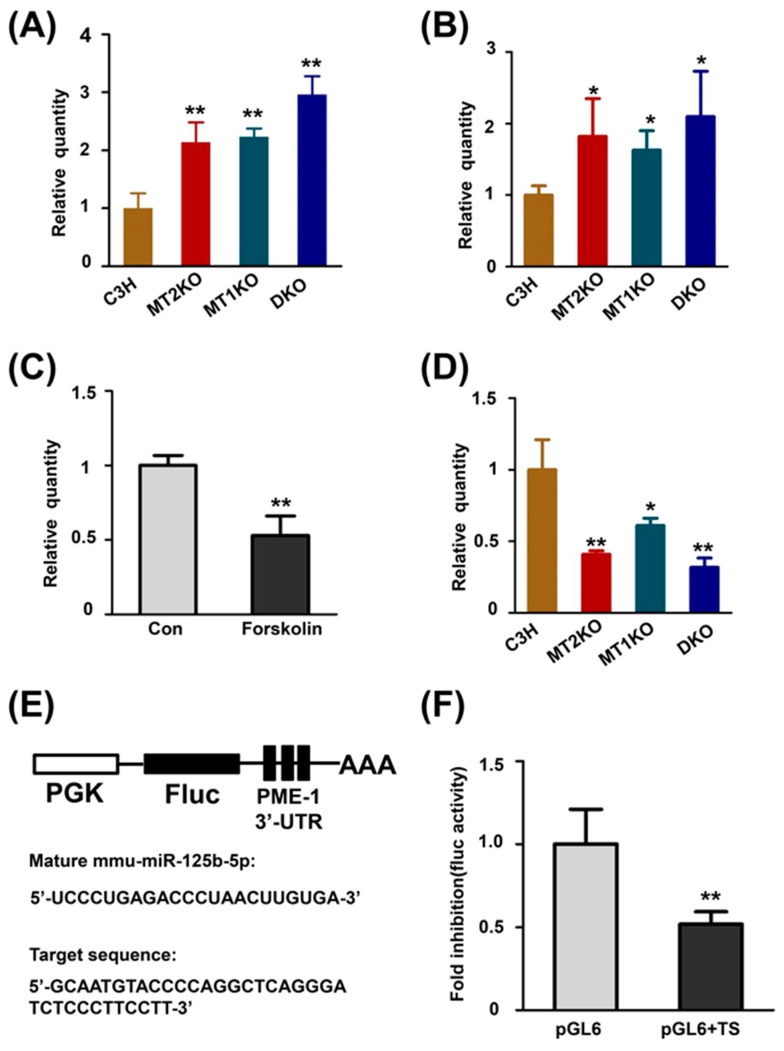
MiR-125b-5p interferes with the translation of PME-1 by binding to its 3′UTR. (**A**) Relative quantity of Ppme1 by RT-PCR in C3H and three transgenic mice. (**B**) Relative quantity of cAMP by ELISA in C3H and three transgenic mice. (**C**) Relative quantity of miR-125b-5p by RT-PCR in HEK293 with or without forskolin. (**D**) Relative quantity of miR-125b-5p by RT-PCR in C3H and three transgenic mice. (**E**) pGL6 plasmid with partial sequence of PME-1′s 3′UTR. PGK, promoter; Fluc: irefly luciferase; AAA: polyA tails. The mature mmu-miR-125b and target sequence are shown at the bottom. (**F**) Fold inhibition of firefly luciferase by miR-125b. TS, target sequence. * *p* < 0.05, compared with C3H; ** *p* < 0.01, compared with C3H. Data are presented as means ± SD.

We speculated that the overexpression of PME-1 may be due to its increased transcription. A significant increase in the level of Ppme1 was found in MT2KO, MT1KO and DKO mice compared with C3H mice by RT-PCR (Figure 5A). To further address the possible role of the melatonin signaling pathway in transgenic mice, we detected the level of cAMP and its downstream target and found significantly increased levels in three transgenic mice groups (Figure 5B) as well as cGMP (Supplementary Figure S4A). It has been reported that cAMP decreases the expression of miR-125b-5p in melanocytes and that miR-125b-5p binds to the 3′UTR of PME-1, as predicted by the TargetScan website (http://www.targetscan.org/vert_72/ accessed on 27 October 2021). To ensure the regulation of miR-125b-5p by cAMP in neurons we detected the level of miR-125b-5p by incubating forskolin in the HEK293 cell line. We found a large decrease in miR-125b-5p due to forskolin (Figure 5C). We detected the expression of miR-125b-5p and found a significant decrease in miR-125b-5p in three transgenic mice group compared with its levels in C3H mice (Figure 5D). Therefore, we constructed a luciferase reporter-gene plasmid (Figure 5E) and compared the expression of firefly luciferase-transfected miR-125b-5p in the pGL6 or pGL6 + TS plasmids. MiR-125b-5p transfection largely decreased the expression of firefly luciferase in pGL6 + TS but not pGL6 (Figure 5F).

### 2.5. Decreased miR-125b-5p Induces Tau Hyperphosphorylation

**Figure 6:**
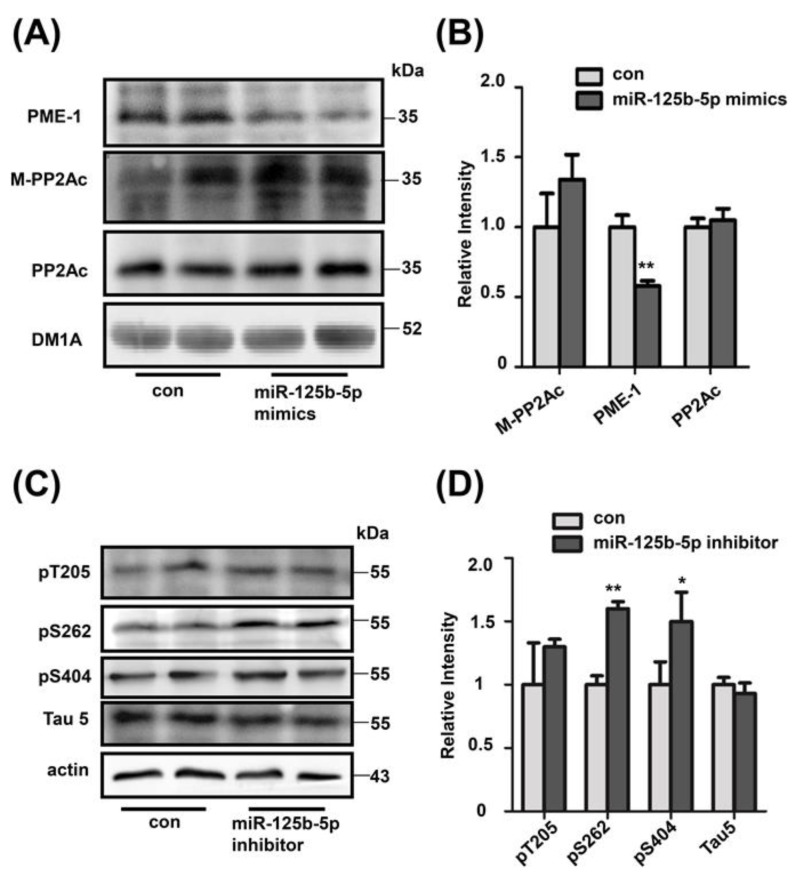
Low expression of miR-125b-5p induces tau hyperphosphorylation by removing the inhibition of PME-1 translation. (**A**) Western blotting of PME-1, m-PP2Ac and PP2Ac. (**B**) Relative intensity of PME-1, m-PP2Ac and PP2Ac. (**C**) Western blotting of pT205, pS262, pS404 and tau 5. (**D**) Relative intensity of pT205, pS262, pS404 and tau 5. * *p* < 0.05, compared with C3H; ** *p* < 0.01, compared with C3H. Data are presented as means ± SD.

To confirm the regulatory role of miR-125b-5p, we transfected miR-125b-5p mimics into HEK293 cells and found decreased expression of PME-1 (Figure 6A,B), which demonstrated that miR-125b-5p could directly inhibit the expression of PME-1 in vitro. Furthermore, by transfecting miR-125b-5p inhibitor into the N2a cell line we observed tau hyperphosphorylation at pS404, pT205 and pS262. (Figure 6C,D). These results demonstrate that the decrease in miR-125b-5p removes the inhibition of PME-1 translation, which induces tau hyperphosphorylation.

## 3. Discussion

**Figure 7:**
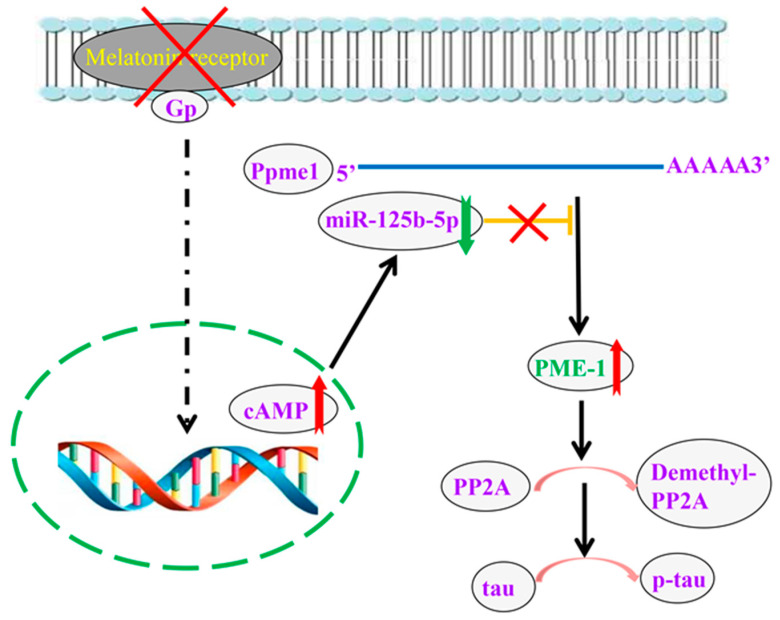
The schematic molecular pathway that links Melatonin receptor knockout and Tau hyperphosphorylation. Melatonin receptor knockout results in a high expression of cAMP in the nucleus, which decreases miR-125b-5p expression. A low level of miR-12b-5p loses the inhibition of Ppme1′s translation. Then, the expression of PME-1 is increased, which causes more PP2A demethylation, further leading to tau hyperphosphorylation.

In this study we confirmed hyperphosphorylated tau at several sites, especially serine 262. The ghostlike neurofibrillary tangles were also observed in melatonin receptor knockout mice. To explore the mechanism, we detected the related kinase and esterase that regulate the phosphorylation of tau, including PP2A, CDK5, GSK-3β, Akt, and ERK. The main reason for tau phosphorylation is the decreased activity of PP2A, partially due to the demethylation of leucine 309 on PP2Ac. Then, we also detected the expression of PME-1 and PPMT, and the results show overexpression PME-1 due to decreased expression of miR-125b-5p caused by the luciferase reporter gene system. Finally, we used miR-125b-5p mimics and inhibitor to ensure the regulation of miR-125b-5p on tau phosphorylation in cell line. The schematic molecular pathway that links melatonin receptor knockout and tau hyperphosphorylation is shown (Figure 7).

The level of melatonin is related to the circadian rhythm, which mainly conducts intracellular signal transduction through melatonin receptors. Melatonin-receptor signaling damage may be involved in sleep disorders [42,43]. Our results indicate that knockout of the melatonin receptor will lead to demethylated PP2A, which leads to tau phosphorylation. Interestingly, the mutation of arginine at position 295 in PP2Ac increases PP2A demethylation accompanying sleep disorders [44]. These results suggest melatonin-receptor signaling pathway plays an important role in the mechanism of sleep disorders. Melatonin is also closely related to AD. Studies have shown that the severity of mental and sleep disorders in AD patients are significantly related to pineal melatonin production and cerebrospinal fluid (CSF) melatonin levels [45]. Our findings indicate that melatonin signaling disorders reduce miR-125b-5p levels. Consistent with our results, the decline in melatonin levels in AD patients is also accompanied by a significant downregulation of hsa-miR-125b-5p expression [46]. Therefore, further study is necessary to explore the role of miR-125b-5p in AD.

Several of randomized trials have been conducted to evaluate the therapeutic effect of melatonin in AD, but the results are not consistent. Some results prove that melatonin can ameliorate the sleep time, night time, and the cognitive function [47]. Other studies suggest there is no significant difference in sleep efficiency and sleep time between the melatonin administration and the negative control [48,49]. The opposite results may be related to the distribution and number of melatonin receptors and also the heterogeneity of the disease. Because melatonin has physiological functions which depend on its receptors, such as antioxidation and combination with neurofibrillary tangles, melatonin receptors are attracting more and more attention as drug targets [50]. Our results demonstrate that the downregulation of melatonin signaling led to the decreased expression of miR-125b-5p. However, overexpression of miR-125b-5p also induces tau hyperphosphorylation and cognitive deficits in Alzheimer’s disease [51]. Hence, further study is needed to uncover its mechanisms.

Previous studies have demonstrated behavioral and functional differences between MT2KO and MT1KO mice. MT1KO mice display depression-like behaviors [52], and MT2KO mice show impaired hippocampal long-term potentiation (LTP) [53]. These different deficits may be related to the different tissue distribution of the melatonin receptors. For instance, researchers reported that expression of melatonin receptor 2 was especially rich in the cortex and hippocampus, and that expression of melatonin receptor 1 was relatively rich in the hypothalamus and thalamus [54]. From the results of Figure 1 and Figure 2, we observe a high phosphorylation of tau in MT1KO mice at the sites of serine 404 and 214, but less phosphorylation of tau at the site of serine 262, compared with MT2KO mice. Meanwhile a higher tangle ratio is observed in MT2KO mice but not in MT1KO mice. We speculate that melatonin receptor 2 may be a critical factor for the formation of neurofibrillary tangles by tau phosphorylated at Ser 262. In AD patients, Ser 262 is hyperphosphorylated in neurofibrillary tangles [55], which strongly inhibits the binding of tau protein to microtubules. Thus, MT2KO mice show a more extensive tangle formation because of higher tau phosphorylation at the site of serine 262 than that of MT1KO mice, while MT1KO mice show a less extensive tangle formation because of lower tau phosphorylation at the site of serine 262 though higher tau hyperphosphorylation at the site of serine 404, 214 and AT8, than that in MT2KO mice.

GSK-3β, CDK5, ERK and Akt are the major kinases that phosphorylate tau, while PP2A is a major phosphatase that dephosphorylates tau [56]. Regarding the decreased activity of those kinases, our results suggested the decreased activity of PP2A was the major cause of tau hyperphosphorylation. PP2A consists of a structural subunit (A), a regulator subunit (B) and a catalytic subunit (C). PP2A activity is regulated by tyrosine 307 and leucine 309 on PP2Ac. From our results, we already knew of an excess phosphorylation of tyrosine 307 and demethylation of leucine 309, which led to dramatically low activity of PP2A in three transgenic mice groups. To further explore the relevant mechanisms, we studied the tyrosine kinase Src which is proven to phosphorylate PP2A at the site of tyrosine 307 [57]. Unfortunately, there was no significant difference among the three transgenic mice (Supplementary Figure S3A,B). PP2A containing a Bα subunit specifically binds to and dephosphorylates tau, which is increased by leucine 309 methylation [58]. Fyn also recognized the same motif of tau, which consequently led to tau hyperphosphorylation [59]. However, our results suggested that the expression of Fyn was not significantly different between the three transgenic mice groups (Supplementary Figure S3A,B). The demethylation of PP2Ac dissociates PP2Ac from the Bα subunit, making it unable to bind tau, thus leading to tau hyperphosphorylation. Our results demonstrated a significant increase in the demethylated PP2Ac subunit, which suggests a primary role in tau phosphorylation.

The levels of the downstream targets of melatonin receptors, such as cAMP and cGMP, were measured. We found a dramatic increase in cAMP in three transgenic mice groups, which was consistent with the other research [60]. These results demonstrate that the melatonin receptor is critical for controlling the generation of cAMP in the neuronal system. Intracellular cAMP level has an extensive impact on biological effect by two classical signaling pathways: the PKA pathway and the EPAC pathway. We supposed that the PKA and EPAC may be different between the three transgenic mice groups. Hence, we detected the changes in PKA and EPAC by Western blot. The PKA molecular composition was obviously changed, but that of EPAC was not (Supplementary Figure S4B), which may partially contribute to tau phosphorylation, especially at the site of Ser214 [61]. cAMP is also suggested to regulate the miR-125b-5p level in pigmented cells [62]. We detected and found a significant decrease in miR-125b-5p levels in three transgenic mice groups. Then, we used TargetScan to predict the miR-125b-5p target gene and found a site on the 3′UTR of PME-1. To validate this prediction, we constructed a luciferase reporter plasmid by inserting the partial 3′UTR sequence of PME-1 into the pGL6 plasmid. MiR-125b-5p significantly inhibited the expression of the luciferase reporter. In brief, our research proves that damage to melatonin-receptor signal transduction causes tau hyperphosphorylation and tangle formation by decreasing miR-125b-5p expression.

From our research, the damage of melatonin-receptor signal transduction plays a critical role in neurodegeneration and tau phosphorylation. Our results could help us to understand the mechanism of neurodegeneration and tauopathy and improve therapeutic strategies.

## 4. Materials and Methods

### 4.1. Animals

Transgenic mice were obtained from Professor D. Weaver at the University of Massachusetts Medical School, and all animals were fed sufficient food and water in a room on a 12-h light–dark cycle at 25 °C. All animal experiments were carried out according to the revised version of the ‘Policies on the Use of Animals and Humans in Neuroscience Research’ approved by the Society for Neuroscience on 1 January 2012. Mice were divided into four groups: C3H mice (C3H), melatonin receptor 2 knockout mice (MT2KO), melatonin receptor 1 knockout mice (MT1KO) and melatonin receptor 1 and 2 knockout mice (DKO). The genetic background of the control group is C3H mice. MT2KO, MT1KO and DKO groups were transgenic mice derived from C3H. The C3H group contained four mice for Western blot and RNA extraction (*n* = 4 per group), two mice for immunochemistry (*n* = 2 per group) and two mice for silver staining (*n* = 2 per group). The mice of all groups were sacrificed at 12 months of age.

### 4.2. Antibodies

A pS404 antibody against tau phosphorylated at Ser 404 was purchased from SAB. An AT8 antibody against human PHF-tau was purchased from Thermo. A pS262 antibody against tau phosphorylated at Ser 262 was obtained from SAB. A pT231 antibody against tau phosphorylated at Thr 231 was obtained from SAB. A pT205 antibody against tau phosphorylated at Thr 205 was purchased from SAB. A Tau5 antibody against the total tau was obtained from Millipore. A GSK-T antibody against the total GSK was from SAB. A GSK-S9 antibody against GSK phosphorylated at Ser 9 was purchased from Cell Signaling. A GSK-Y216 antibody against GSK phosphorylated at Tyr 216 was purchased from Santa Cruz. An Akt 308 antibody against Akt phosphorylated at Thr 308 was obtained from Abcam. An Akt 473 antibody against Akt phosphorylated at Ser 473 was purchased from Cell Signaling. An Akt-T antibody against the total Akt was obtained from Cell Signaling. A P35 antibody against P35 was from Santa Cruz. A CDK5 antibody against cyclin-dependent kinase 5 was purchased from Santa Cruz. A *p*-ERK antibody against phosphorylated ERK was purchased from Cell Signaling. An ERK antibody against external stimulus regulated kinase was purchased from Cell Signaling. A PME-1 antibody against protein phosphatase methylesterase was purchased from Upstate. A PPMT antibody against protein carboxyl methyltransferase was purchased from Upstate. All antibodies were used at a concentration of 1:1000 for Western blotting and 1:200 for immunohistochemistry. Antirabbit IRDye and antimouse IRDye were purchased from Li-Cor Biosciences (Lincoln, NE, USA). A BCA kit was purchased from Pierce (Rockford, 1 L, Rockford, IL, USA). A kit to determine the quantity of cAMP was from YaJi. A kit to detect the activity of PP2A was from Promega.

### 4.3. Western Blot

The hippocampus was removed from the brain and homogenized in tissue homogenate buffer. The sample was added to a one-third volume of sample buffer containing 8% sodium dodecyl sulfate (SDS), 200 mM Tris, and 40% glycerol, and was boiled for 10 min. The lysate was centrifuged at 12,000× *g* for 10 min at 4 °C, and the supernatant was stored at −80 °C. The supernatant protein concentration was detected using the BCA method. The same amount of protein in each lane was separated by SDS-polyacrylamide gel electrophoresis (10%) and transferred to a nitrocellulose filter membrane. Membranes containing the protein were blocked with 3% bovine serum albumin and incubated with antibody at 4 °C overnight. Then, the membrane was incubated with goat antimouse or goat antirabbit IgG conjugated to IRDye (1:10,000, Li-Cor Biosciences) for 1 h at room temperature. The membrane was visualized by an Odyssey imaging system.

### 4.4. Cell Culture and Treatments

A mouse neuroblastoma cell line, N2a, was cultured in complete media containing DMEM (11965084, Gibco, Carlsbad, CA, USA) with 10% fetal bovine serum (FBS) (C0232, Beyotime, Shanghai, China). Cells were maintained at 37 °C, 5% CO_2_ and 95% humidified air. Cells were seeded in a six-well plate and treated with forskolin (S1612, Beyotime, Shanghai, China) for 24 h and then harvested for microRNA extraction or Western blot.

### 4.5. Immunohistochemistry

Each mouse was anesthetized with 6% chloral hydrate, rapidly perfused with 300 mL saline and then perfused with 500 mL 4% paraformaldehyde for 2 h. The brain was removed from the skull and sliced into 30 μm slices with a vibratome (Leica, VT1000S, Germany) after postfixation in the same fixative in a 50 mL centrifuge tube. Each brain slice was soaked in PBS containing 0.5% Triton and 0.3% H_2_O_2_ to remove endogenous hydrogen peroxidase and then blocked with 3% BSA for 30 min. Next, the slice was incubated with primary antibody (1:200) for 48 h at 4 °C. Then, the slice was incubated with biotin-labeled secondary antibody for 1 h in a 37 °C oven and stained with a diaminobenzidine tetrachloride system (ZSGB-BIO, 9032, Beijing, China). Images were taken with a light microscope (Olympus BX60, Tokyo, Japan).

### 4.6. PP2A Activity Assay

Homogenates were prepared with a homogenizer in buffer (50 mM Tris-HCl, 100 mM EDTA, 0.5 mM PMSF, 150 mM NaCl), and a Sephadex G-25 slurry was prepared. Under frozen conditions, the Sephadex G-25 slurry was shaken six times every 30 min. The Sephadex G-25 slurry was centrifuged to remove the Sephadex storage buffer. A phosphate storage buffer was loaded onto the resin and centrifuged at 600× *g* and 4 °C for 10 min. The protein sample was loaded and centrifuged at 600× *g* and 4 °C for 10 min. Then, the BCA method was used to detect the protein concentration. The sample was diluted to 1 µg/µL, and reaction buffer and storage buffer were added at 30 °C for 30 min. Finally, a dye solution was used to terminate the reaction after 20 min. The OD was measured at 630 nm.

### 4.7. Bielschowsky Silver Staining and Analysis

We placed the slices into a 10% AgNO_3_ solution at 37 °C in the dark for 30 min. The slices were washed in PBS for 5 min, 3 times. We incubated the slices in 10% formaldehyde for 5 min. Then, we incubated the slices in silver–ammonia ethanol solution for 5 min and then in 8% formaldehyde.

### 4.8. cAMP ELISA

A 100 µL sample was added to each tube at 37 °C for 2 h. The liquid was discarded, and 100 µL biotin-labeled antibody working solution (1:100) was added at 37 °C for 1 h. The liquid was discarded, and the sample was washed 3 times with 350 µL wash buffer. One hundred microliters of a horseradish peroxidase-labeled avidin working solution was added at 37 °C for 1 h. The liquid was discarded, and the sample was washed 3 times with 350 µL wash buffer. Ninety microliters of the substrate solution were added at 37 °C for no longer than 30 min while avoiding light. Fifty microliters of a termination solution were added to terminate the reaction. The optical density (OD) at 450 nm was read with a microplate reader within 15 min.

### 4.9. RNA Isolation and Real-Time PCR

Total RNA was isolated from cell or frozen mouse tissues using TRIzol reagent (R0016, Beyotime, Shanghai, China). cDNA was generated from 10–100 ng of total RNA (D7168S, Beyotime, Shanghai, China). qPCRs were conducted with SYRB Green qRT-PCR kit according to the manufacturer’s protocol (D7260, Beyotime, Shanghai, China). Samples were normalized to β-actin. MicroRNA was collected by using a Rapid Tissue Cell MicroRNA Extraction Kit (B1802, HaiGene, Harbin, China) and then reverse-transcribed into cDNA using the TaqMan microRNA Reverse Transcription Kit (D1802, HaiGene, Harbin, China) following the manufacturer’s instructions. Real-time PCR was performed using the HG TaqMan miRNA qPCR Kit (TAP01394, HaiGene, Harbin, China) to detect the quantity of the mmu-miR-125b-5p. Samples were normalized to snoU6. The results were calculated by the 2^−^^ΔΔCt^ method 19246619. All primers were synthetized by Qingke Company, Beijing, China, and listed in Table 1.

### 4.10. Luciferase-Reporter Plasmid Construction and Transfection

The basic pGL6 plasmid (D2106, Beyotime, Shanghai, China) was purchased from Beyotime Biotechnology. A plasmid named pGL6 + TS was constructed by cloning the target sequence to the 3′UTR of pGL6 plasmid by QingKe Company. The target sequence was 5′-GCAATGTACCCCAGGCTCAGGGATCTCCCTTCCTT-3′. Mmu-miR-125b-5p was synthetized from QingKe. The pGL6 and pGL6 + TS plasmids were both cotransfected with mmu-miR-125b-5p into N2a cells by lipo8000 (C0533, Beyotime, Shanghai, China). A total of 10^5^ cells were evaluated per well in 24-well plates. Twenty-four hours later, the cells were collected, and the firefly luciferase activity was detected by a firefly luciferase-reporter gene detection kit (RG005, Beyotime, Shanghai, China).

### 4.11. Statistical Analysis

For statistical analysis, differences between groups were tested by ANOVA followed by the LSD post hoc test using SPSS 17.0. To compare single differences, the significance of the differences between means was determined by the *t*-test. *p* < 0.05 indicated statistical significance.

## Figures and Tables

**Table 1:** List of RT-qPCR oligonucleotides used and assay performances.

Gene Name	RT Oligonucleotide	qPCR Forward Primer	qPCR Reverse Primer
MiR-125b-5p	CACCGTTCCCCGCCGTCGGTGTCACAA	GCCTCCCTGAGACCCTA	CCGTCGGTGTCACAAGTTAG
SnoU6	CACCGTTCCCCGCCGTCGGTGCTTCTC	CTCGCTTCGGCAGCA	GCCGTCGGTGCTTCTCTGT
Ppme1		TCAAATGTCTTCCCAGGCTCAG	CCACCGCTCGCTATGGCTAA
β-actin		GTCGTACCACAGGCATTGTGTGG	GCAATGCCTGGGTACATGGTGG

## Data Availability

The data can be obtained from the corresponding author upon request.

## Supplementary Materials

The following are available online at www.mdpi.com/article/10.3390/ijms222111850/s1.

## Abbreviations

melatonin receptor-2 knockout, MT2KO; melatonin receptor-1 knockout, MT1KO; melatonin receptor 1 and 2 knockout, DKO; protein phosphatase 2A, PP2A; enzyme-linked immunosorbent assay, ELISA; protein phosphatase methylesterase-1, PME-1; glycogen synthase kinase-3β, GSK-3β; cyclin-dependent kinase 5, CDK5; protein kinase B, Akt; cyclic adenosine monophosphate, cAMP; cAMP-responsive element binding protein, CREB; protein kinase A, PKA; cyclic guanosine monophosphate, cGMP; protein kinase C, PKC; suprachiasmatic nucleus, SCN; Alzheimer’s disease, AD; catalytic subunit of PP2A, PP2Ac; PP2A-methyltransferase, PPMT.
