# Supplementary figures and images for: Hyperphosphorylation of Tau Due to the Interference of Protein Phosphatase Methylesterase-1 Overexpression by MiR-125b-5p in Melatonin Receptor Knockout Mice
Source: Int J Mol Sci. 2021 Oct 31;22(21):11850. doi: 10.3390/ijms222111850 (PMC8611649; doi:10.3390/ijms222111850)

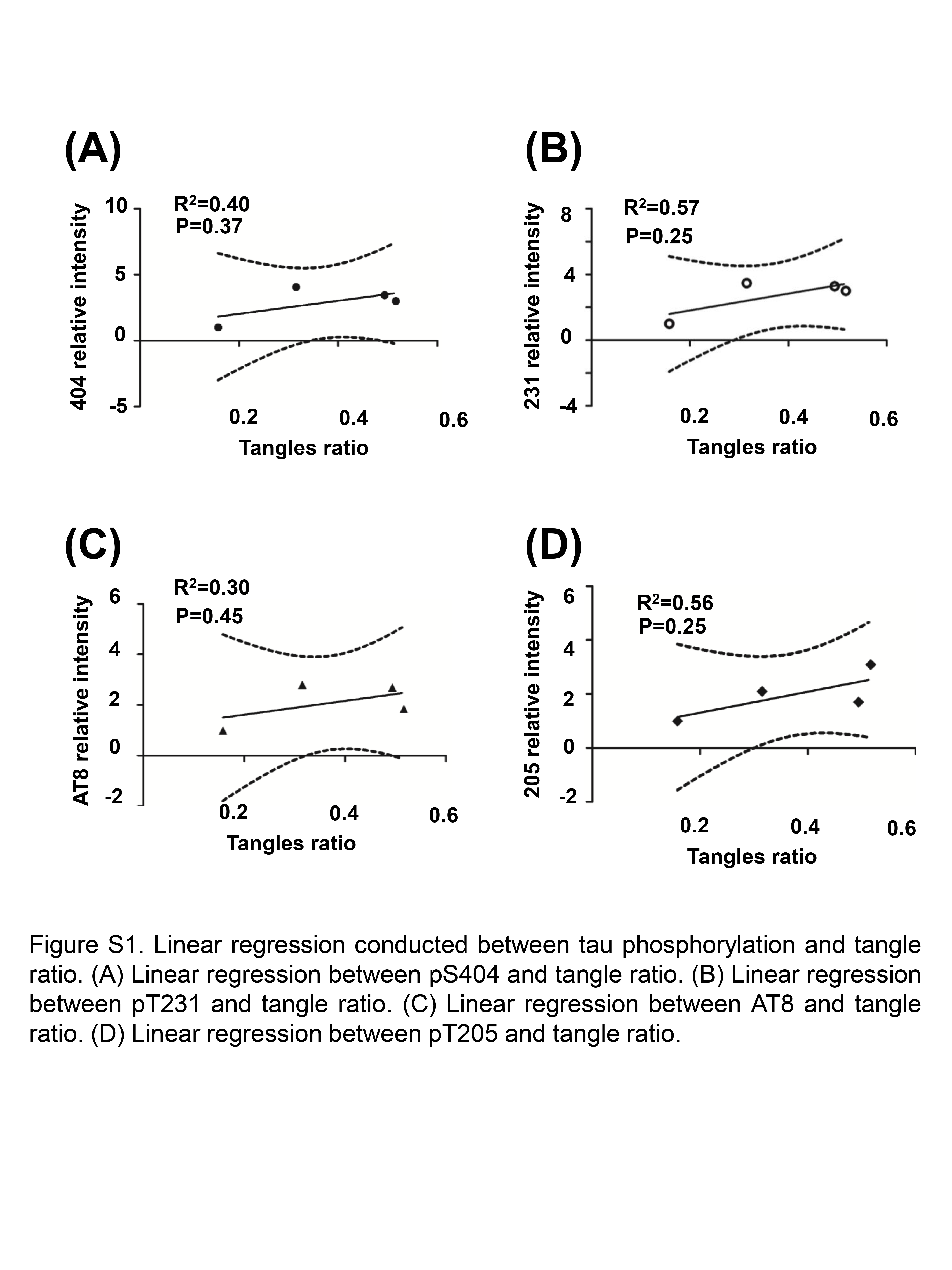

Supplement: Supplementary file 1 [file ijms-22-11850-s001.zip › ijms-1402539 sup revised/Supplementary files/Figure S1.tif]

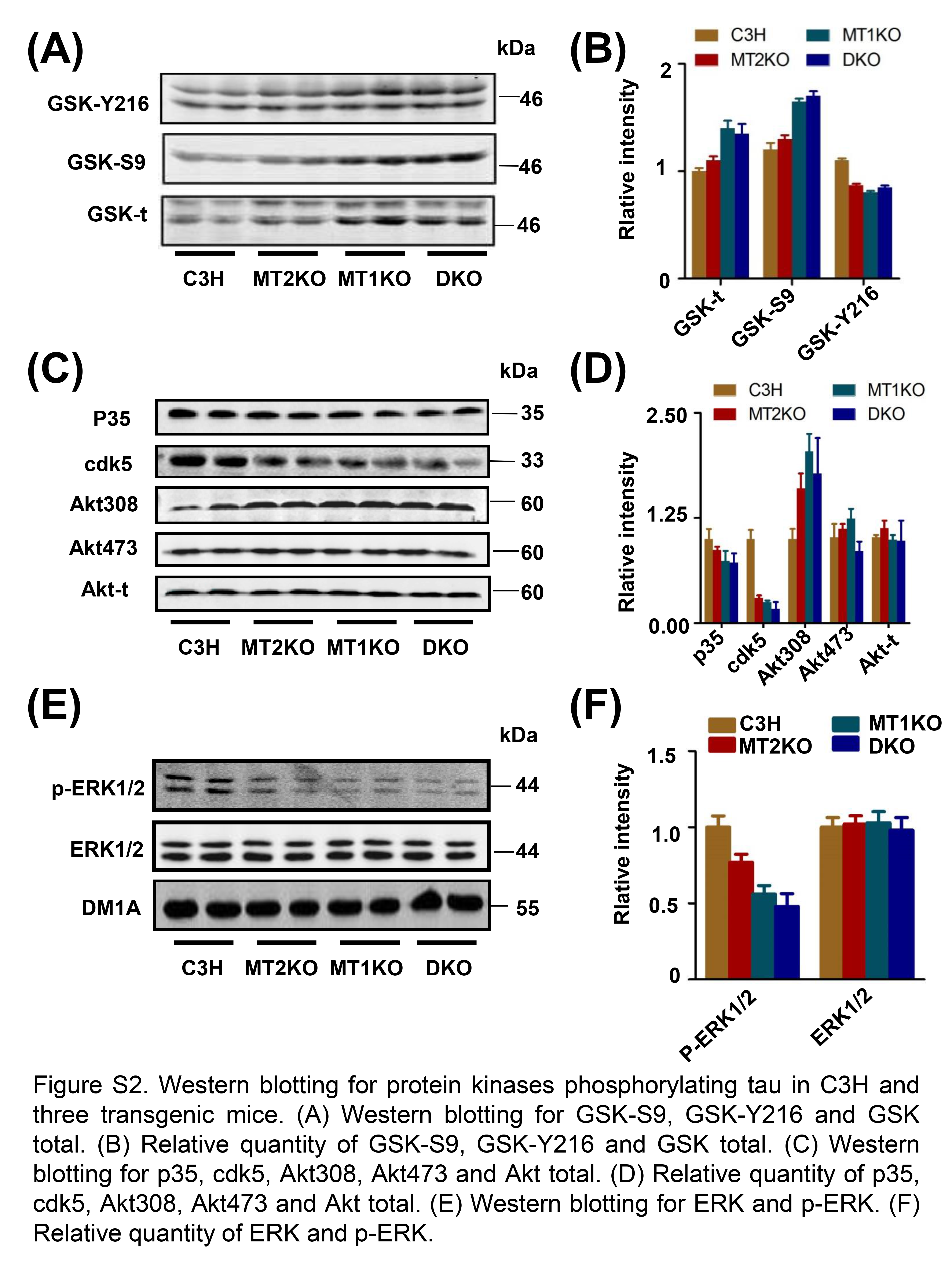

Supplement: Supplementary file 1 [file ijms-22-11850-s001.zip › ijms-1402539 sup revised/Supplementary files/Figure S2.tif]

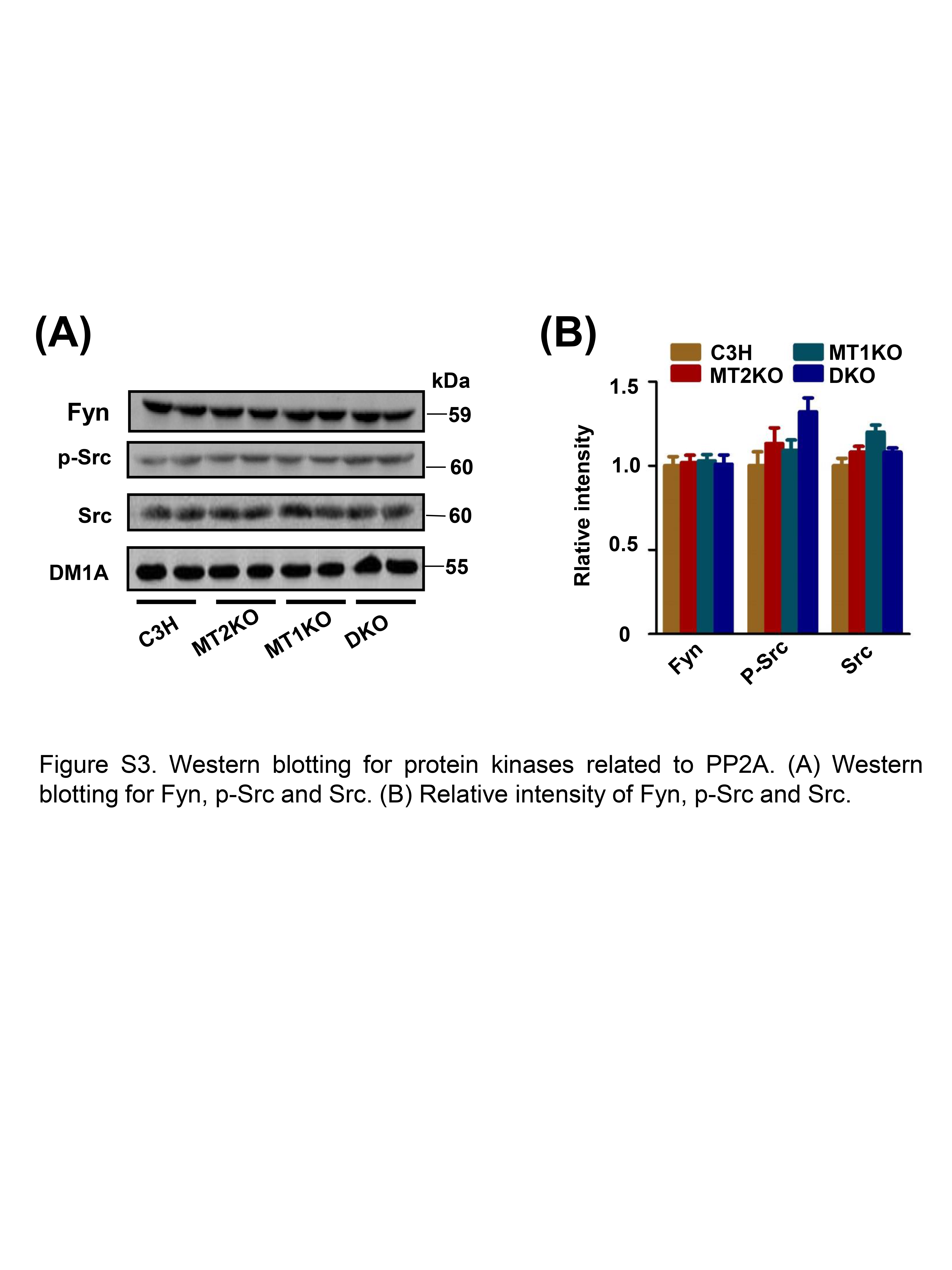

Supplement: Supplementary file 1 [file ijms-22-11850-s001.zip › ijms-1402539 sup revised/Supplementary files/Figure S3.tif]

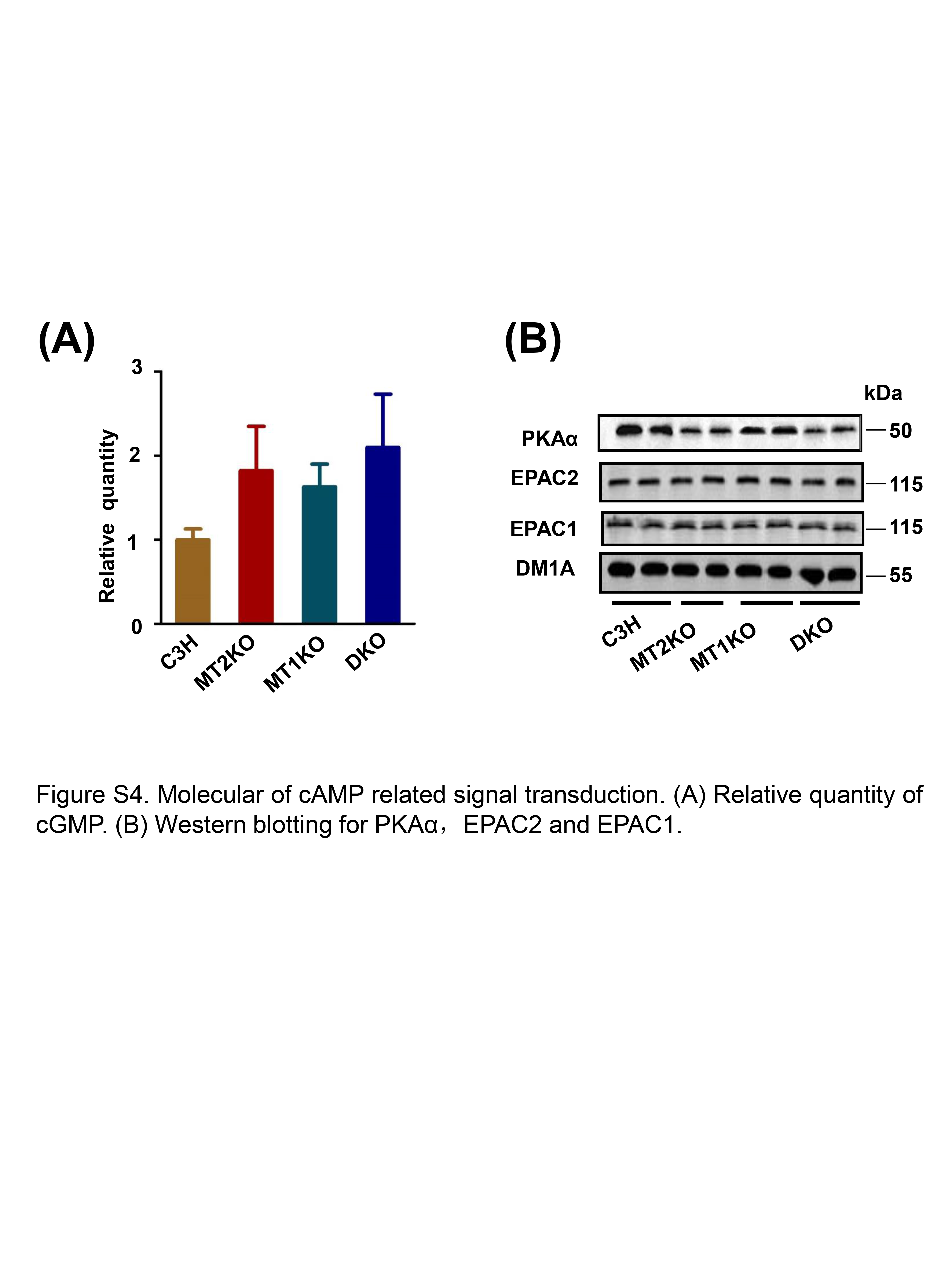

Supplement: Supplementary file 1 [file ijms-22-11850-s001.zip › ijms-1402539 sup revised/Supplementary files/Figure S4.tif]
